# Supplementary material for: Construction and application of a novel urban knowledge model with extended historical and cultural semantics
Source: PLoS One. 2025 Sep 22;20(9):e0331170. doi: 10.1371/journal.pone.0331170 (PMC12453240; doi:10.1371/journal.pone.0331170)
Supplement: Attachment — Submitted filename: Response to Reviewers.docx [file pone.0331170.s002.docx]

Dear Editor and Reviewers:

Thank you for your thorough review of our manuscript and for providing such valuable and constructive feedback. We sincerely appreciate the time and effort you dedicated to evaluating our work. Based on your insightful suggestions, we have carefully revised the manuscript to address all comments. Below is a summary of the key revisions made:

1. We have expanded the background discussion to provide a more rigorous foundation for the study’s motivation, clearly delineating the significance of our work.
2. The research questions, hypotheses, and contributions have been further clarified. The paper now explicitly presents a methodological framework spanning modeling to application, with empirical validation using a case study from Guangzhou.
3. The scope of the literature review has been broadened to include a more systematic synthesis of prior studies, with additional comparisons and critical analysis to better position our work within the existing scholarship.
4. The discussion now more tightly aligns with the research objectives, elaborating on how the study achieves its proposed goals while exploring its broader implications and potential applications.
5. The conclusion has been augmented with a deeper reflection on the study’s limitations, particularly regarding data imbalance, spatial unit selection, and model generalizability, providing avenues for future research.

We believe these revisions have significantly strengthened the manuscript and are grateful for the opportunity to improve it. Please do not hesitate to contact us if further clarifications or adjustments are needed.

Thank you again for your time and consideration.

*Note: In this document, the reviewers' original comments are displayed in* ***orange text****, while the authors' responses are in black.*

# Reviewer 1

1. **In the 5th line, a direct transition to machine learning has been made, artificial intelligence should be mentioned before.**

With regard to the comment on line 5 (assumed to refer to line 35 in the full manuscript, i.e., the fifth line of Introduction Section 2), we acknowledge that the original transition from urban historical semantics projects to machine learning applications in urban informatics appeared abrupt.

Both urban historical semantics projects and urban informatics research are underpinned by artificial intelligence technologies, the former aligns with symbolic AI approaches, while the latter employs connectionist AI techniques. We have incorporated this clarification in the revised manuscript, see *line 37-51*.

2. **Contributions are not explained concretely and clearly**

The previous version might have caused some misunderstanding regarding the description of this paper's contributions. In the Introduction section in this revised version, we have clarified the contributions as follows: *Our contribution is the development of a comprehensive methodological framework that encompasses the construction of a knowledge graph integrating both contemporary and historical dimensions, the training of embedding models, and their application scenarios. The viability of this framework is empirically demonstrated through a case study conducted in Guangzhou. （see line 63-67）*

3. **Literature studies on the subject are missing**

We sincerely appreciate the reviewer’s insightful observation regarding the literature review on the subject. The reviewer rightly points out the need to contextualize our work within existing research on urban semantic extraction and structure both historical and contemporary.

However, we acknowledge that few studies explicitly bridge these two dimensions. This gap motivates our work’s core contribution which propose a framework to integrate historical-cultural and contemporary semantics into an urban knowledge graph.

Our framework is not developed in isolation. We integrated approaches from Reference[31],[33] and [92]. Reference [92] provided the technical framework and methodological reference for extracting contemporary urban semantics. Reference [31] and [33] inspired the extraction of historical-cultural semantics in this study.

In this revision, we have supplemented the literature review with additional fields to better compare and position the research presented in this paper. We have included open-source projects on historical place semantics, as well as attempts by governments and administrative bodies to use digital means to promote and showcase local cultural content. Please see "Extracting historical and cultural semantics in the digitization of cultural heritage" and "research gaps" section.

We hope this revision addresses the reviewer’s concern and more clearly establishes our work’s novelty.

4. **Explanations of the figures are missing**

We have carefully reviewed the figure captions and the main body of manuscript, revising some figure captions (e.g., Figures 1, 5, 6, 8, and 9) to more clearly describe the content of the figures. Additionally, for some figures (e.g., Figure 2), we have added further explanations in the main text to enhance the interpretation of their content.

5. **Where is the dataset? Was it shared as an open link or are there any benchmark datasets on this subject?**

Thank you for your reminder regarding the dataset availability. We have now uploaded our research dataset. It is accessible via **https://github.com/DaqunW/UrbanKG_GZ_case**

6. **Why were graph-based structures not preferred? Like graph neural network**

We sincerely appreciate the reviewer’s insightful question regarding our choice of knowledge graph embedding (KGE) methods. The reviewer rightly points out that graph neural networks (GNNs) are indeed well-suited for processing graph-structured data and could also be applied to KGE. However, after careful consideration, we opted for the ConvE model rather than GNN-based approaches for the following reasons:

1. Our study primarily relies on link prediction as a key function of knowledge graph embedding. While GNNs excel at generating node/entity embeddings, they do not inherently perform relation prediction—instead, they require additional decoder design to achieve this. In contrast, ConvE is specifically designed for triple-based link prediction, making it a more direct and efficient choice for our task.
2. Existing literature supports that ConvE outperforms GNN-based models in certain scenarios. Dettmers T et al (2018) demonstrated that ConvE achieves superior performance on benchmark datasets like FB15k-237, particularly in link prediction tasks, while maintaining higher parameter efficiency compared to GNNs.
3. While some GNN variants show advantages in tasks requiring joint modeling of nodes and relations (e.g., node classification or graph classification), ConvE remains highly competitive for simpler link prediction tasks, which aligns with our focus.

We acknowledge that our initial submission did not sufficiently justify the model selection. In response, we have expanded the discussion in Section *Knowledge graph embedding* to explicitly compare ConvE with other alternatives. We hope this clarification addresses the reviewer’s concern.

7. **How was the batch size or optimization algorithm selection made in model training? Was early stopping done? In Figure 6 (Model overfitting is visible).**

We sincerely appreciate the reviewer's valuable questions regarding the model training setup and potential overfitting. In this study, we empirically set the batch size to 100. While overfitting generally harms generalization, its effect is mitigated in our specific context for three reasons:

1. This study prioritizes a novel pipeline (from data processing → KG embedding → downstream applications) rather than building a universally generalizable model. The Guangzhou case study serves as a proof of concept to demonstrate the framework’s feasibility and utility. This study does not propose a generalized model applicable to any city.
2. For our application scenarios, higher prediction accuracy is prioritized over generalization. Overfitting here helps the model capture Guangzhou-specific patterns, which aligns with our goal of maximizing accuracy for this city’s unique data.
3. Urban knowledge graphs are highly city-dependent due to geographical, historical, and cultural variations. Thus, even a "perfectly" generalized model would require training for new cities. Each city's model should train independently to ensure accurate results.

We have included an analysis of the model’s generalization limitations in the *Discussion* section (see *line 611-616*), clarifying its scope and potential improvements for broader applications.

8. **How were the evaluation metrics selected, are there any other metrics?**

During experiments, we measured multiple metrics including Training Loss, MR (Mean Rank), MRR (Mean Reciprocal Rank), and Hits@1/10. These indices are commonly used in KGE tasks. *Training Loss* is a fundamental indicator of model convergence. *MRR + Hits@n* is a widely adopted combination that balances accuracy (via MRR) and robustness (via Hits@n). We ultimately retained Training Loss, MRR, and Hits@10 in the manuscript. Because MR and MRR, as well as Hits@1 and Hits@10, exhibited highly correlated trends in our results. Hits@10 provides a more lenient and informative measure of model performance compared to Hits@1, while MRR captures the overall ranking quality.

9. **There is no transition to Subject integrity in the entire article**

We sincerely appreciate the reviewer's valuable feedback regarding the paper's transition to its core themes. In this revised version, we have thoroughly restructured both the '*Discussion'* and *'Conclusion*' sections to better articulate and reinforce our research focus.

In the discussion section, we systematically address the two research hypotheses proposed in the Introduction. First, we demonstrate the effectiveness of constructing an urban knowledge graph that integrates both historical and contemporary semantics as a holistic digital infrastructure infrastructure. Second, we validate the proposed application scenarios' efficacy and potential value, supported by detailed interpretations of the model's output results.

The conclusion section has been strengthened to Reaffirm the study's contributions in:

1. Addressing the challenges of historical-cultural place semantics
2. Explicitly connect our work to UNESCO's policy initiatives
3. Clearly position the significance of our methodological approach within the research gap

We believe these revisions have substantially improved the paper's focus and clarity.

10. **No references Neo4j**

We sincerely appreciate the reviewer's valuable observation. In response, we have supplemented section 'Graph database evaluation and visualization' with a reference to a study that employs Neo4j for processing large-scale SNS data while evaluating its performance. This addition serves to demonstrate Neo4j's general applicability in handling relational data. See line *314*.

11. **Figure 9 is a word cloud shape?**

Yes. In Section '*Mining potentially similar places and entities*', we let the model to output all entities that are semantically proximate to the spatial grid cells within Guang-Shang District. The model calculates pairwise similarity between entities and ranks the results in descending order. For clearer visualization, we presented spatial entities in Figure 8, and displayed non-spatial entities in Figure 9. The font size of each term in Figure 9 corresponds to their similarity score.

12. **There is no connection between quantitative and qualitative results.**

The qualitative findings in this study are derived from quantitative computations, including link prediction and entity similarity calculation. Specifically:

1. In the first application scenario, both the spatial entities (Figure 8) and non-spatial entities (Figure 9) were obtained through entity similarity calculations performed on spatial grid units within the Guang-Shang District.
2. The destination recommendation results in the second application scenario were generated through multiple head/tail entity prediction operations.
3. The third application scenario follows a similar approach to the first one, where recommended locations for new branches of time-honored brands were determined by calculating entity similarity based on existing time-honored brand locations.
4. The fourth scenario involved head/tail entity predictions for specified relationship types (population movement), followed by aggregated statistical analysis to generate visitor demographic profiles.

# Reviewer 2

1. **Strengthen Theoretical Engagement and Conceptual Framing: While the manuscript effectively adopts the HUL framework and introduces knowledge graph technologies, it would benefit from deeper theoretical engagement with literature on digital heritage, place-based semantics, and spatial humanities. Concepts such as "cultural memory," "urban narrative," and "semantic place theory" could provide a richer interpretive lens and help the study speak more broadly to international academic debates.**

We sincerely appreciate the reviewer's valuable suggestions regarding theoretical foundations. In response, we have substantially restructured the “*Introduction*” section (see line *1-36*) to incorporate deeper theoretical engagement. The revised introduction now establishes its theoretical foundation through *Semantic Place Theory*, systematically explicating the concept of place semantics and its significance. In this version, we position *historical and cultural semantics* as a critical dimension of place semantics, currently facing dual challenges of gradual dissolution and public oblivion. Beyond the *Historic Urban Landscape (HUL)* framework, we have incorporated UNESCO's *digital heritage* policy documents to strengthen policy-oriented motivation. We believe this restructuring provides stronger theoretical anchoring.

2. **Clarify Research Objectives and Contributions in the Introduction: The introduction presents a compelling case for the relevance of integrating historical and contemporary semantics, but the structure would be improved by clearly enumerating the study's research questions and hypotheses. The objectives are somewhat embedded in the narrative; making them more explicit would help orient the reader.**

We are most grateful to the reviewer for raising this pertinent observation. Our initial submission didn't adequately situate the research problem, objectives, and contributions, which regrettably led to understandings.

In this revised version, we have thoroughly restructured the 'Introduction' section to clearly set out the research question, hypotheses and objectives, see line *52-67*.

Our research question is:

【How can digital technologies construct an effective bridge between historical-cultural and contemporary place semantics】

Our research hypotheses are:

1. Resolving this question requires a holistic digital infrastructure capable of integrating the comprehensive semantics of urban places, encompassing both contemporary and historical-cultural dimensions.
2. Furthermore, practical implementation pathways must be outlined to maximise the potential of such an infrastructure.

Our contribution is:

We develop a comprehensive methodological framework that encompasses the construction of a knowledge graph integrating both contemporary and historical dimensions, and their application scenarios. The viability of this framework is empirically demonstrated through a case study conducted in Guangzhou.

3. **Enhance Methodological Transparency: The methodology is highly complex, yet several stages (e.g., the grounded theory tagging system for cultural types, the threshold selection for OD and workplace flows, and the justification for temporal segmentation) are described in a fragmented or insufficiently detailed manner. A tabulated summary of the workflow steps, supported by schematic diagrams, would improve replicability and transparency.**

We sincerely appreciate the reviewer's invaluable suggestion. In the original version, section '*Methodology*' proceeded directly to explain individual steps without providing an overview of the complete workflow. To address this, we have implemented the following improvements (see line *164-177*):

1. Figure 2 revisions. We add a comprehensive workflow diagram on the right, optimize the graphical presentation for greater clarity and intuitiveness, and aligned each step name in the figure with corresponding subsection headings.
2. Expansion of 'Methodology' section. A general framework of the methodology is introduced at the beginning, while more detailed technical content appears in the subsequent subsections.

These modifications have been implemented in the revised manuscript to provide better methodological transparency and logical flow. We believe these improvements effectively address the reviewer's concern while maintaining the paper's technical rigor.

4. **Refine Language and Presentation: The manuscript would benefit significantly from English language editing. Several sections are overly long, with inconsistent terminology and occasional grammatical errors. Improving the clarity and coherence of the writing will greatly enhance the manuscript’s readability and impact.**

We sincerely appreciate the reviewer's careful reading. In this revision, I have carefully checked the entire manuscript to ensure consistency in the use of terminology. For instance, terms such as "place semantics", "contemporary semantics" and "historical and cultural semantics" (sometimes write as historical-cultural semantics) are now used consistently throughout the text. Additionally, I have thoroughly reviewed the grammar and made necessary corrections to address any issues that may have been present.

I acknowledge the 'Data and Methods' section's extended length. we have deliberately maintained its integrity to detail the full pipeline from UrbanKG construction, training, to implementation. The sections titled '*Experimental evaluation findings and analysis*' and '*Application scenarios*' essentially constitute the '*Results*' part of the paper. The '*Experimental evaluation findings and analysis*' section is dedicated to interpreting the metrics to assess the effectiveness of the model and its training. Meanwhile, the '*Application scenarios*' section illustrates how the model can be utilised in various scenarios and elucidates its output results. I think this structure will enable readers to follow the research process and findings in a logical and coherent manner.

5. **Critically Reflect on the Limitations and Ontological Biases: The paper acknowledges the imbalance between contemporary and historical data but does not sufficiently explore the implications. A more reflective discussion of biases introduced by the dominance of contemporary data sources, the manual processing of historical information, and the assumptions embedded in spatial units (e.g., 100m x 100m grid cells) would strengthen the epistemological rigor.**

The digital era has enabled the acquisition of high-precision, large-scale contemporary urban spatiotemporal data. However, historical data requires additional processing (digitization, structuring, etc.) of archival materials and historical documents before utilization, making it inherently challenging to achieve comparable volume to contemporary datasets.

To mitigate this imbalance, our study employs a flexible model-switching approach between M1, M2 and M3:

- For purely historical-dimension semantics (e.g., Scenario 1), the historically-trained subgraph model (M2) is preferred due to its superior historical relationship learning capability
- Contemporary-dimensional analyses (e.g., Scenarios 2 and 4) utilize either M1 or M3
- Integrated historical-contemporary applications employ M3

However, the persistent data imbalance may still bias M3 (considering both contemporary and historical-cultural semantics) toward contemporary patterns during training potentially compromising historical relationship prediction accuracy. To address this issue, it is necessary in future research to assign a higher weight to the historical dimension triplets within the loss function of the M3 model.

The discussion regarding the imbalance between contemporary and historical data has been added to both the '*Discussion*' section and '*Conclusion*' section. See line *584-593* and *612-623.*

This research utilizes 100×100m spatial grids as basic analytical units for data integration, following established conventions in geographic studies. Areas that exhibit notable spatial variability may be consolidated into single grid cells, potentially leading to discrepancies between the aggregated grid-level characteristics and finer-scale observations. These discrepancies predominantly affect contemporary data aggregation. In the aggregation of historical data, this study establishes one-to-multiple correspondence relationships for each grid unit to comprehensively capture all cultural semantics present within the grid, as the historical-cultural data is discontinuous. The discussion regarding the potential implications of employing 100m×100m grids as fundamental research units has been incorporated into the “*Discussion*” section. Please see line *630-638*.

6. **Expand the Discussion of Applicability and Transferability: The model is applied to Guangzhou, a rich and data-accessible case. It would be useful to address how UrbanKG might be adapted to other urban contexts, especially those with less structured data or more contested heritage landscapes. A comparative or scenario-based reflection could broaden the scope of the study’s contributions.**

We sincerely appreciate the reviewer's valuable suggestions for improvement. This study aims to propose a generalizable methodological framework, with its core workflow comprising: *"spatial data allocation -> schema definition -> knowledge graph construction -> model training -> scenario applications"*, using the Guangzhou case study to validate its feasibility. It is important to clarify that our primary objective is not to develop a highly universal and generalizable model, but rather to establish a replicable methodological approach. These points have been explicitly restated in the Contribution subsection of the revised Introduction (see Lines *63-67*).

Regarding application in data-scarce urban contexts, I think contemporary and historical-cultural patterns are both likely to demonstrate significant variations across cities. Consequently, direct model transferability cannot be reliably guaranteed. For implementation in new urban contexts, we recommend either acquiring city-specfic data, or utilizing data from cities with demonstrably similar historical-cultural profiles. A discussion of model generalizability considerations has been added to the manuscript (see line *639-644*).

7. **Deepen the Analytical Interpretation of Results: Much of the evaluation, particularly in Sections 3.1–3.3, focuses on performance metrics. While this is important, a stronger interpretive narrative is needed to connect these technical outcomes to substantive urban issues. For example, how might high semantic similarity between grids translate into planning insights, or how do the model's predictions align (or not) with existing policy frameworks?**

We sincerely appreciate the reviewer's constructive suggestion. In response, we have expanded the discussion regarding the correlation between model predictions and real-world urban environments in Section "*Embedding Model Validity Testing*" (Lines *388-398* and *413-415*). The relevant content provides further interpretation regarding the consistency between model predictions and real-world urban environments and some insights for urban planning applications, particularly in job-housing balance analysis.

# Reviewer 3

1. **The two core objectives—building a systematic urban knowledge model and promoting public awareness—are only partially achieved. The first is well addressed; the second lacks implementation, validation, or user engagement.**

We are grateful to the reviewer for highlighting this important issue regarding the articulation of our research objectives. Our initial submission didn't adequately situate the research contributions, which regrettably led to understandings. In this revised version, we have carefully refined the statement of objectives in the “Introduction” section to explicitly clarify that:

*Our contribution is the development of a comprehensive methodological framework that encompasses the construction of a knowledge graph integrating both contemporary and historical dimensions, the training of embedding models, and their application scenarios. The viability of this framework is empirically demonstrated through a case study conducted in Guangzhou. (Line 63-67)*

It should be noted that the present study focuses specifically on establishing the methodological feasibility of this approach, while further research is required to experimentally and empirically assess its implementation effects, such as whether it enhances the public's understanding of historical culture.

2. **The research gap is real but overstated. The paper overlooks existing public-facing historical KGs like World Historical Gazetteer (WHG), Linked Places, and Recogito, which already support semantic historical exploration. A more nuanced comparison would better position the contribution.**

We are most grateful for the reviewer's valuable references to the WHG, Recogito and Linked Places projects, which we recognise as exemplary digital humanities initiatives in historical-cultural dimension of place semantics. In response to this suggestion, we have substantially expanded the Section "*Extracting Historical and Cultural Semantics in the Digitisation of Cultural Heritage*" to provide a detailed review of these foundational works and other related studies or projects on historical and cultural semantics. These works also encompass the idea of interconnecting spatial locations through historical semantics, which is highly consistent with my concepts.

The distinctive contribution of our study lies specifically in its innovative integration of historical-cultural semantics with contemporary place semantics. We maintain that only through simultaneous consideration of both temporal dimensions can identify meaningful spatial connections across time, then develop practical implementations for bringing historical meaning into modern urban contexts.

We hope this clarification adequately addresses the reviewer's concerns regarding the study's contributions.

3. **The four application scenarios are promising but remain simulated. No real-world deployment, user testing, or institutional collaboration is shown.**

We sincerely appreciate the reviewer's valuable suggestions regarding our study's real-world deployment, user testing and possible institutional collaboration. We acknowledge that these are limitations of our current work, as they extend beyond the primary scope of this paper.

We would like to clarify the core objective of our work is to propose a comprehensive framework that encompasses the construction of the holistic urban knowledge model, and propose its application scenarios. And through the case study of Guangzhou, we demonstrate that it is feasible to achieve this goal by leveraging existing data and the technical pathways outlined in this paper.

In summary, this study remains at the experimental stage, with its principal contribution being the establishment of technical viability using existing data and our proposed methodology.

In response to the model deployment, user testing and institutional collaboration mentioned by reviewer's comments, we envisage that these could be realised in the future through the development of a web-based interface. The application value of the model could then be quantified through user surveys and expert evaluations.

4. **Claims about planning support and cultural sustainability are made but not operationalized with metrics or institutional partnerships.**

We are most grateful to the reviewer for raising these significant observations. We acknowledge that the current discussion regarding planning support and cultural sustainability remains principally grounded in theoretical justification and case study validation, without yet incorporating formal institutional collaboration or standardised metrics for quantitative assessment.

In the 'Discussion' section, we have elaborated on the potential value of our four application scenarios (see line *527-550*):

1. Scenario 1 (Reasoning about places with similar cultural semantics) can facilitate public discovery of cultural connections between urban locations, serving an educational function, and provides empirical foundations for cultural heritage conservation, such as themed heritage trail planning for sites with high semantic correlation, coordinated promotional strategies, and cultural branding initiatives.
2. Scenario 2 (Developing multiple cross-references to recommend destinations) enables personalised cultural tourism recommendation.
3. Scenario 3 (Historic establishment location analysis) supports the continuity of urban historical context within contemporary spatial patterns.
4. Scenario 4 (Cultural site visitor profiling) assists cultural authorities in identifying cultural heritage sites with insufficient current recognition and enables them to adjust publicity strategies in accordance with the target audience.

This systematic analysis demonstrates how our framework could theoretically contribute to both planning support systems and cultural sustainability objectives. We hope this detailed explanation helps alleviate the reviewer's concerns regarding the current lack of specific implementation metrics and institutional collaboration plans in our study.

5. **Public engagement is claimed but not measured. There are no user-facing interfaces, behavioral data, or feedback mechanisms.**

We extend our sincere gratitude for the reviewer's meticulous and rigorous evaluation of our manuscript. Regarding the concern about "Public engagement is not measured," upon revisiting the text, we recognize that certain expressions in the Introduction and Application Scenarios sections may have caused misunderstanding.

In this revised version, we have explicitly clarified that the study's primary objective is to propose a comprehensive methodological framework spanning from urban knowledge model construction to practical application, and demonstrate its feasibility through the Guangzhou case study, using existing data and our proposed technical approach. So our current research represents an experimental-stage, proof-of-concept exploration focused on validating the scientific rigor of methodology and exploring technical feasibility.

We fully acknowledge that empirical evaluation of public engagement effectiveness requires further dedicated research. It necessitates further dedicated investigation. In subsequent research phases, we propose to: (1) develop dedicated web-based interfaces serving as participatory platforms for public engagement, and (2) implement mixed-method evaluation frameworks incorporating user studies, expert assessments, and systematic behavioral data collection to establish robust feedback mechanisms. This multidimensional approach will enable quantitative assessment of urban knowledge graphs' practical efficacy in enhancing public historical-cultural awareness. (3) Furthermore, we intend to pursue institutional collaborations to facilitate the translation of this research from laboratory validation to real-world implementation scenarios.

6. **Historical data imbalance is acknowledged but not addressed—triples are unweighted, and manual data curation is not algorithmically compensated.**

Digital technologies have enabled unprecedented access to high-resolution, large-scale spatiotemporal data for modern urban analysis. By contrast, historical datasets require extensive preprocessing of archival materials—including digitization, structural annotation, and semantic enrichment—thereby creating inherent asymmetries in data volume and quality across temporal dimensions. To address this temporal data disparity, we implement an adaptive modeling framework that strategically employs M1-M3 based on analytical requirements.

However, the data imbalance may still introduce bias in the M3 model's training process, predisposing it towards learning patterns from contemporary data at the expense of historical relationship prediction accuracy. To mitigate this limitation, future research should implement weighted loss functions in the M3 model that assign higher weights to historical-dimension triplets during training.

The discussion regarding the imbalance between contemporary and historical data has been added to both the '*Discussion*' section and '*Conclusion*' section. See line *584-593* and *612-623.*

7. **The model is trained and tested solely on Guangzhou. No replication or transferability to other cities is explored.**

We sincerely appreciate the reviewer's constructive feedback. We acknowledge that this study exclusively employed Guangzhou's data for model training and testing, without conducting transferability validation across other cities. It stems from the following considerations:

1. The study aims to establish a generalizable methodological framework with the core workflow: "spatial data allocation → schema definition → knowledge graph construction → model training → scenario applications". The Guangzhou case serves as proof-of-concept validation. We don't aim to develop a universally applicable model. This clarification has been explicitly articulated in the revised '*Introduction*' section (see Lines *63-67*).
2. Significant divergences exist in both historical-cultural and contemporary semantic patterns across cities. Direct model transfer would likely compromise predictive performance. We believe that customized models need to be developed using the proprietary data of target cities, or through transfer learning from cities with highly similar place semantics patterns.

These considerations have been thoroughly discussed in Section '*Concusion and future work*' (see lines *639-644*). We believe this explanation adequately addresses the reviewer's concerns.

8. **The use of behavioral data (e.g., Weibo check-ins) is not accompanied by any discussion of ethics, anonymization, or review protocols.**

We are most grateful to the reviewer for raising this important consideration regarding our data sources. As this study primarily constitutes a methodological exploration, we have used only standardised, readily available, and secure datasets throughout our research.

The weibo check-in data were procured from a commercial third-party provider. The raw dataset contained no personally identifiable information, only included anonymised spatial-temporal records (location coordinates and timestamps). Furthermore, in this study, the Weibo check-in locations were aggregated and tallied to represent the number of check-ins within each study unit, thereby serving the vitality level evaluation. So there is no possibility of any privacy leakage.

9. **Data transparency is insufficient—no sample triples, pipeline diagrams, or data access options are provided, limiting reproducibility.**

We sincerely appreciate the reviewer's valuable feedback regarding data transparency and reproducibility. In this revision, we have uploaded all triple data and complete codebase (see **https://github.com/DaqunW/UrbanKG_GZ_case**). In order to conserve space, we have presented both the data sources and the entities in Table 2, with each entity corresponding to its source data and the method of data processing.

Our handling of the data essentially follows the workflow:

1. Spatial location verification

2. Aggregation to 100m×100m grid units

3. Schema-based conversion to triple format

The contemporary dimension data were commercially acquired from third-party provider. And historical and cultural data were manually extracted from textual sources through entity recognition and annotation processes.

10. **The use of ConvE is not justified or compared with other models. No explanation is given for choosing it over alternatives like TransE, RotatE, or CompGCN, despite the spatiotemporal nature of the data.**

We are most grateful to the reviewer for raising this insightful observation. In the original manuscript, we regrettably omitted a thorough justification for selecting the ConvE model as our knowledge graph embedding approach. In response, we have substantially expanded the methodological rationale in the revised version (Lines *274-285*).

ConvE has been empirically proven to outperform traditional knowledge graph embedding models such as TransE, DistMult, and ComplEx in link prediction tasks (Dettmers T et al, 2018). Although RotatE (Sun Z et al, 2019; Luo Z et al, 2020) and CompGCN (Vashishth S et al, 2019) exhibit superior performance when handling large-scale datasets and complex relations, this study ultimately adopts ConvE due to the single-city scale of the dataset and the consideration of model lightweight requirements.

The terms “space” and “place” are used interchangeably without defining their conceptual distinction, which is important in urban semantics.

11. **The terms “space” and “place” are used interchangeably without defining their conceptual distinction, which is important in urban semantics.**

We sincerely appreciate the reviewer's thorough and constructive evaluation of our manuscript. In response to the valuable feedback, we have implemented the following improvements in this revised version. (1) We have addressed all specific concerns raised in the review through substantive modifications. (2) We have also conducted a comprehensive linguistic review to rectify any grammatical inaccuracies and to ensure terminological consistency throughout the text. (3) We have maintained the standardized usage of "place semantics" as the preferred term, with the sole exception being the foundational conceptual differentiation between "space" and "place" presented in the begin of "*Introduction*" section.

12. **Minor Observations:**

| Line 186: “functions contains” → should be corrected to “functions that include”. | This error has been rectified in the revised version of the text |
| --- | --- |
| Line 28: “aims to bridge” → should be “aim to bridge” (plural subject). | This error has been rectified in the revised version of the text |
| Line 454: Duplicate word — “the the” should be corrected. | This error has been rectified in the revised version of the text |
| Line 503: Clarify “limits regression tasks” → “limits support for regression tasks.” | The original text has been revised to clarify its meaning and now modified to: "consequently limiting its application to regression-based analytical tasks." |
| Line 422: Missing cross-reference — “Section ?” should be specified. | This error has been corrected in the current version. The issue stemmed from a LaTeX syntax error. In this revised manuscript, since individual sections are not numbered, we have instead directly referenced the section headings. |
| Figure 2: Spelling error — “sematics” → should be “semantics.” | This error has been rectified in the revised version of Figure 2. |
| Figure 5: Nearly identical to Figure 2 and possibly redundant; consider merging or removing. | This version features an updated Figure 2 that presents each methodological step with enhanced clarity and visual intuitiveness, while ensuring strict correspondence between these steps and their respective subheadings in the Methods section. A supplementary workflow diagram has been incorporated into the right panel to provide additional procedural context. Furthermore, the revised figure has been thoroughly redesigned to eliminate any potential visual or conceptual similarities with Figure 5, ensuring complete graphical distinctiveness. Therefore, Figure 5 has been retained. |
| Figure 2: Mentions a “Memory Platform” that is never explained in the text—should be clarified or removed. | This version has updated Figure 2 that presents each methodological step with enhanced clarity and visual intuitiveness, while ensuring strict correspondence between these steps and their respective subheadings in the “Methodology” section. |
| Several phrases (e.g., “public-facing cultural semantics,” “cultural tagging system,” “WorkedAt”) use non-standard or unclear terminology and should be refined for clarity. | In the revised version, the term "public-facing cultural semantics" has been removed entirely, with "historical and cultural semantics" retained as the sole technical terminology. Clarifications have been added upon their first occurrence for both the "cultural tagging system" and "WorkedAt" predicate. |
| The term “Time-Honored Brand” is central to one application scenario but is not defined; a brief explanation would aid understanding. | An explanatory note on the term "Time-Honored Brand" has been incorporated into the "Time-Honored Brand Site Selection" section of the revised manuscript. |
| The term “semanticization” should be replaced with the more conventional “semantic enrichment.” | This error has been rectified in the revised version |
| “Panoramic knowledge” is used metaphorically and repeatedly but is not a standard term. It should be replaced with “comprehensive” or “holistic,” or defined clearly if retained. | The revised version has eliminated this particular expression, replacing it with the more precise formulation: "knowledge incorporating both historical and contemporary semantic dimensions." |

# Reviewer 4

1. **The methodological flowchart (Figure 2) needs to be revised. The text in the figure, especially on the top right, is not legible. I suggest the author(s) use a more legible font type and color.**

We sincerely appreciate the reviewers' constructive suggestions for improvement. In this revised version, Figure 2 has been updated to improve clarity and readability. All texts elements in Figure 2, including labels and annotations, have been standardized to ensure clear legibility.

In the original version, Figure 5 was directly utilized in Figure 2 to illustrate our ontological schema. In this revised iteration, we have modified the presentation approach by employing concrete examples to demonstrate both the schema and triple instances of the knowledge graph.

2. **Can the author(s) provide citation for the grounded theory workflow used in developing a cultural tagging system. This will strengthen the credibility of the approach.**

We sincerely appreciate the reviewer's valuable suggestion. In this revised version, we have incorporated reference to grounded theory methodology （see line *201*), which provides a comprehensive workflow for conducting grounded theory research. Furthermore, we have expanded the discussion of the cultural tagging system (Lines *201-204*) to more explicitly illustrate how it draws upon and adapts key principles from grounded theory approaches.

3. **I recommend that the author(s) should provide citation for the knowledge graph embedding (KGE) technology that was used in obtaining the vectorized representations of the triples. I believe many studies have used the same approach. Also, it would be great if the author(s) could justify the choice of the ConvE model for knowledge graph embedding over alternatives like TransE or DistMult.**

We are grateful to the reviewer for raising this insightful observation. Upon reflection, we acknowledge that the original submission did not adequately justify our choice of the ConvE architecture for knowledge graph embedding. To address this, we have significantly enhanced the methodological justification in the revised manuscript (Lines *274-285*), providing a more comprehensive comparative analysis.

The selection of ConvE was based on several key considerations:

1. ConvE has demonstrated superior performance over conventional approaches (TransE, DistMult and ComplEx) for link prediction tasks (Dettmers et al, 2018).
2. While other models like RotatE (Sun et al., 2019; Luo et al., 2020) and CompGCN (Vashishth et al., 2019) show advantages with large-scale datasets and complex relations, ConvE demonstrates particular suitability for our research context due to: (1) its computational efficiency matching our single-city dataset scale, (2) robust performance on fundamental relationship prediction tasks, and (3) lightweight architecture requirements.

In summary, ConvE achieves an optimal balance between prediction performance and training efficiency, making it our model of choice for knowledge graph embedding (KGE) in this study.

4. **The author should double check the first part of the sentence in line 411-413 and make proper reference of the appropriate section. Also in line 422. The author must have omitted the Section number.**

We sincerely appreciate the reviewer's meticulous review. The identified issue stemmed from a LaTeX formatting error, which has now been corrected in this revised version. Additionally, we have conducted a comprehensive check to ensure the resolution of similar potential issues throughout the manuscript.

# Reviewer 5

**The paper "Construction and Application of a Novel Urban Knowledge Model with Extended Historical and Cultural Semantics" is technically sound and written in a very standard fashion, addressing a very interesting issue. The analysis has been performed rigorously. No comments to the authors.**

We sincerely appreciate the reviewer's kind words regarding the technical soundness and scholarly presentation of our work. Your positive evaluation motivates our continued research efforts in this field.

# Reviewer 6

1. **…Nevertheless, a more critical discussion of the accuracy and limitations of the manual annotation process of historical data would be desirable…**

Thanks to the review's valuable suggestions. To ensure that readers can understand both the methodology of this paper and the reliability as well as potential limitations of data processing, we have added a discussion on the accuracy and limitations of the manual annotation process for historical data in the "*Unstructured Historical Data Processing*" section, lines *208-217*.

The extraction of attribute information in historical data processing relies heavily on manual interpretation. The raw unstructured historical materials used in this study typically contain explicit spatial information (e.g., locations of historical places or buildings), historical figures, and events elements, making these features relatively straightforward to identify. However, the classification of cultural types based on the cultural tagging system involves subjective judgment, potentially leading to discrepancies among different annotators. To ensure the rationality and standardization of cultural tag assignments, this study implemented a quality control procedure consisting of three stages: initial parallel annotation by multiple annotators, followed by cross-validation, and finalized by a dedicated reviewer for verification.

2. **… as well as a more explicit quantitative assessment of the impact of integrating historical dimensions on the predictive performance of the combined model (M3). Although standard metrics such as MRR and Hits@n are mentioned, the manuscript could improve if a deeper discussion of model generalization outside of the Guangzhou case study was undertaken.**

We sincerely appreciate the reviewers' meticulous reading and constructive comments on this manuscript.

The predictive performance evaluation of Model M3 has been presented and analyzed in the "Embedding Model Performance Evaluation" section. Please refer to Figure 6 and Table 5 for details.

The discussion regarding the generalizability of the model to cities beyond Guangzhou has been supplemented in the "Conclusion and Future Work" section (see lines *639–644*). We argue that, on one hand, the semantic patterns of historical and contemporary dimensions vary significantly across cities, making it difficult to ensure satisfactory performance when directly applying a model trained on Guangzhou data to other cities. If the model is to be applied elsewhere, it would require data from the target city or highly similar proxy data. On the other hand, our primary objective is to propose a generalizable methodological framework, centered on the core workflow of "data spatial localization → schema definition → knowledge graph construction → model training → scenario application," with the Guangzhou case serving as a validation of its feasibility. This study does not aim to develop a highly universal and generalizable model.

3. **Another minor issue is about Fig.1, which must show the study area at national level, because the non-Chinese readers could not know where the study has been undertaken.**

We appreciate the reviewers' valuable suggestions for improvement, and Figure 1 has been revised accordingly based on these comments.

# Reviewer 7

1. **It is suggested that the literature review could be expanded to also focus on how planners and policy makers are contributing to research in the subject area.**

We primarily focus on how digital technologies (such as augmented reality and deep mapping) enhance urban historical spatial narratives, thereby expanding the contributions of planners and policymakers in the research domain. The revised version includes additions to the "Extracting Historical and Cultural Semantics in the Digitization of Cultural Heritage" section of the literature review chapter (see lines *96–109* for details).

2. **Adding an academic contribution map to section “Discussions” could be effective in revealing how research contributes to the field.**

We sincerely appreciate the reviewer's valuable suggestion regarding the inclusion of an academic contribution map to better visualize this study's impact. While we have not incorporated a dedicated contribution map in the current version, we have substantially revised both the Discussion and Conclusion sections to more explicitly articulate the study's academic contributions to the field. Specifically:

1. The Discussion section revisits the two-step research approach initially proposed in the Introduction section, followed by a detailed analysis of how effectively this study has accomplished these steps.

2. At the beginning of the Conclusion section, we have revisited the research background, UNESCO's policy advocation, and existing gaps in the field, aiming to more clearly position the significance of this study.

We hope that the revisions made to the Discussion and Conclusion sections will help to alleviate the reviewer's concerns regarding the positioning of the paper's academic contributions.
